# Bond Strengths of Silorane- and Methacrylate-Based Composites to Various Underlying Materials

**DOI:** 10.1155/2014/782090

**Published:** 2014-05-07

**Authors:** Sezin Ozer, Emine Sen Tunc, Nihan Gonulol

**Affiliations:** ^1^Departments of Pediatric Dentistry, Faculty of Dentistry, Ondokuz Mayıs University, Atakum, 55139 Samsun, Turkey; ^2^Department of Restorative Dentistry, Faculty of Dentistry, Ondokuz Mayıs University, Atakum, 55139 Samsun, Turkey

## Abstract

*Objective*. To evaluate shear bond strength (SBS) values of a methacrylate (FZ 250) and a silorane-based (FS) resin composite to various underlying materials. * Materials and Methods*. A total of 80 samples were prepared with four different underlying materials; a flowable (FLC) and a bulk-fill flowable composite (BFC), and a conventional (CGIC) and resin modified glass-ionomer cement (RMGIC). These underlying materials were laminated plus to methacrylate or silorane-based resin composites (*n* = 10). To evaluate the specimens SBS values were evaluated with a universal testing machine (cross-head speed; 1.0 mm/min). Statistical comparisons were carried out using two-way ANOVA and Tukey's post hoc test with a significance level of *P* < 0.05. * Results*. SBS values for FZ250 were significantly higher than for FS for all of the underlying materials tested (*P* < 0.05). SBS values of FZ250 to BFC were significantly higher than to all other materials (*P* < 0.05), whereas SBS values of FS did not vary significantly according to underlying material (*P* > 0.05). * Conclusion*. The use of FS in conjunction with any of the tested materials showed lower SBS than the FZ 250. Also, new low elastic modulus liner BFC presented slightly good interfacial adhesion so, the usage of BFC as an underlying material may be preferable for FZ 250.

## 1. Introduction

Composite resins are the most common tooth-colored restorative materials used for aesthetic purposes [1]. However, their polymerization shrinkage remains a major drawback to their clinical success as dental restorative materials [2, 3]. Numerous clinical strategies have been suggested and technologies developed with the aim of reducing polymerization shrinkage. Clinical approaches have focused on the modification of curing light source and irradiation sequence and incremental placement of resin composite in posterior teeth [4–6]. Also layering techniques for resin composites that contain the use of low elastic modulus liners under the resin composite [7–10] such as flowable composites and glass ionomer cements have been found to be of great interest [11]. Technological approaches have focused on amounts and types of matrix monomer and filler, initiator level, and the addition of nonbonding microparticles [12].

Recently, dental composites have been developed based on silorane chemistry [13]. These composites contain oxirane and siloxane molecules that are polymerized through a ring-opening mechanism that results in a lower level of polymerization shrinkage relative to other polymerization mechanisms [14, 15]. In addition, their hydrophobicity of siloxane helps to reduce polymerization shrinkage [16]. However, while the low levels of polymerization shrinkage of silorane-based composite have been noted, other issues have been raised, namely, problems in cavity wall adaptation and curing in deep cavities [17]. These limitations suggest that, in clinical situations, the use of some type of material beneath the silorane composite may be beneficial [14, 18]. In the dental industry's ongoing search for materials with improved properties, a new generation of flowable composites, known as “bulk-fill flowable composites,” has been introduced to the dental market. These new products have higher filler contents and are said to have better mechanical properties than earlier composites and are thus recommended for large posterior restorations [19]. By promoting light transmittance, bulk-fill flowable composites have been reported to enable a depth of cure in excess of 4 mm, thereby reducing polymerization shrinkage of laminated composite materials [20], simplifying the filling procedure, and saving precious chair time [21–23].

Generally posterior composite resin restorations placed without a liner show good longevity [24, 25]. However, in terms of clinical practice, dentists may choose layering techniques that combine positive properties of several restorative materials. For the success of layering technique there should be a reasonable bond between two materials [26]. But there is limited knowledge about the bonding properties of combining different materials, especially with regard to silorane-based resin composites and bulk-fill flowable composites. Therefore, this study aimed to evaluate SBS values of a silorane-based resin composite (Filtek Silorane, FS) and a methacrylate-based resin composite (Filtek Z250, FZ250) to a bulk-flowable composite, a regular flowable composite, a conventional glass ionomer cement, and a resin modified glass ionomer cement. The null hypotheses tested were as follows. (1) The bonding performance of a silorane-based resin composite does not differ from that of a methacrylate-based resin composite. (2) The SBS values of resin composites are not affected by the type of underlying material used.

## 2. Materials and Methods

### 2.1. Test Materials

The materials used in the study (including lot numbers and manufacturers' information) are listed in Table 1. Composite materials included a methacrylate-based composite (FZ250, Filtek Z250) and a silorane-based composite (FS, Filtek Silorane), and underlying materials included a regular flowable composite (FLC, ÆliteFlo), a bulk-fill flowable composite (BFC, SDR flow), a conventional glass ionomer cement (CGIC, Riva self-cure), and a resin modified glass ionomer cement (RMGIC, Fuji II LC).

### 2.2. Preparation of Specimens

Underlying material specimens were prepared using plastic molds drilled with holes 10 ± 0.1 mm in diameter and 1 ± 0.1 mm in depth. For each underlying material, 20 specimens were prepared, for a total of 80 specimens. During setting, tops and bottoms of the molds were covered with cellulose acetate strips and glass microscope slides, and hand pressure was applied to produce a smooth surface. For the CGIC specimens, the assembly was held in place for 10 min. For the light-cured test materials, the filled molds were cured on both sides and in different positions for a total of 40 seconds using an LED curing unit (Elipar Free Light II, 3M/ESPE, St. Paul, MN, USA; light intensity: 1000 mV/cm^2^). Following polymerization, specimens were removed from molds, and any excess material was removed by gentle grinding on both sides with 600-grit silicon carbide paper (Phoenix Beta, Buehler, Germany) until flat and equal surfaces were obtained. Debris was removed with a dust blower.

Prior to placement of composites, Clearfil SE bond was used with FZ250, and the Filtek Silorane system adhesive bond was applied with the silorane composite, as recommended by the manufacturer.

Following initial preparations, specimens of each group of underlying material were divided into 2 subgroups according to the composite to be used in lamination. A 2 mm high cylindrical polyethylene tube with an internal diameter of approximately 8 mm was placed on the surface of each underlying specimen, the tube was filled with composite (either FZ250 or FS), and all samples (except the self-cure samples) were polymerized for 20 s using an LED curing unit, with all procedures carried out at room temperature. Light-cured samples were then stored in an incubator at 37°C and 100% humidity for 24 h. The self-cure CGIC specimens were stored at 37°C for 1 h before being immersed in distilled water at 37°C for the next 23 hours [27]. All specimens were then thermocycled between 5°C and 55°C for 500 cycles using a dwell time of 10 s and a transfer time of 30 s between each bath.

### 2.3. Shear Bond Strength Testing

Specimens were subjected to shear bond strength (SBS) testing by placing them in a universal testing machine (LRX Lloyd Instruments, Ametek Inc., Leicester, UK) and removing the tubes with a sharp blade at a crosshead speed of 1.0 mm/min. Load at debonding was recorded in Newtons, and MPa was calculated by dividing this value by the bonded area (mm^2^).

Debonded specimens were examined under a stereomicroscope (Nikon SMZ 1500, Tokyo, Japan) at ×25 magnification, and failure modes were classified as either cohesive failure (fracture inside the composite resin or underlying material), adhesive failure (fracture within the bonding interface), or mixed failure (a combination of cohesive and adhesive failures) [28].

### 2.4. Scanning Electron Microscopy (SEM)

Specimens were gold-sputtered under a high vacuum and examined by SEM at ×25 and ×100 magnification (JEOL, JSM-6400, Tokyo, Japan).

### 2.5. Statistical Analysis

SBS values were expressed as mean ± standard deviation. Statistical analysis was performed using SPSS for Windows, Version 12.0.1 (SPSS Inc., Chicago, IL, USA). Normal distribution of variables was determined by Levene's test, and SBS values were analyzed using two-way ANOVA. Multiple comparisons were performed using Tukey's post hoc test, with a significance level of *P* < 0.05.

## 3. Results

SBS values are given in Table 2. SBS values of FZ250 were significantly higher than SBS values of FS for all underlying test materials (*P* < 0.05). Among FZ250 specimens, the SBS value for BFC was significantly higher than all other underlying materials (*P* < 0.05), whereas no other significant differences were found (*P* > 0.05). Among the FS specimens, CGIC had the highest SBS value of all the underlying materials tested; however, none of the differences in SBS values for any of the underlying materials were statistically significant (*P* > 0.05).

**Figure 1:**
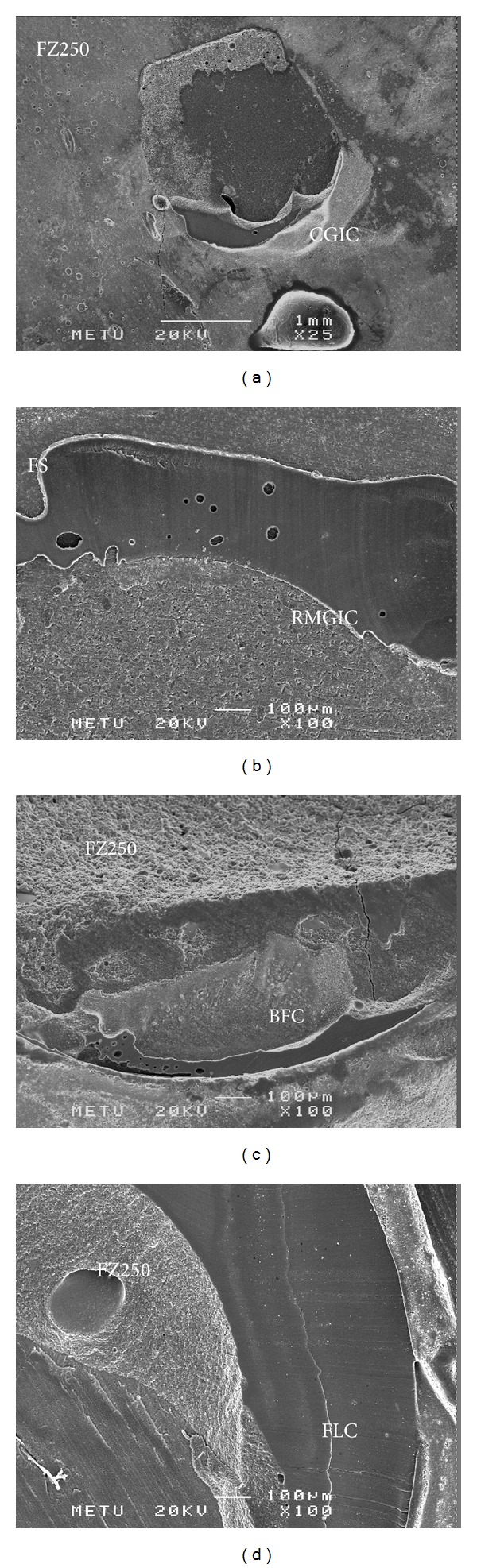
(a) Representative cohesive fracture showing good interlocking adhesion between FZ250 and CGIC. (b) Representative adhesive fracture (FS and RMGIC). (c) Mixed fracture (FZ250 and BFC). Adhesive failure observed in the underlying material along with cohesive failure in the bulk-filled composite. (d) Mixed fracture (FZ250 and FLC). Adhesive and cohesive failure observed in FZ250 and in the flowable composite.

Fracture analysis results are given in Table 2. Mixed and cohesive failures were the most common failure modes for the FZ250 group, whereas adhesive failure was most common for the FS group. SEM views of different groups were shown in Figures 1(a)–1(d).

## 4. Discussion

Although resin composites have shown great improvement, the clinicians still prefer to apply layering techniques that aimed to combine the favorable properties of different materials in a single restoration [11, 29]. Thus, by decreasing the bulk amount, of resin used, this technique can also reduce the detrimental effect of polymerization shrinkage, which may result in microleakage and marginal gap [30]. In addition, it acts as stress absorbing layer between the shrinking composite and dentine [31]. If the quality of interfacial adaptation between two materials could be improved it was assumed that the durability of layered resin restorations may be increased [32].

This study showed that the SBS values of FS to underlying materials were lower than those of FZ250 (*P* ≤ 0.05), with adhesive failure, the most common failure mode for FS. The lower bonding performance of FS could be attributed to its low reactivity and to low surface wettability of the intermediate resin adhesive. The silorane adhesive system is based on methacrylate chemistry (technical information from 3M-ESPE) [33, 34], which allows conventional methacrylate-based composites to bond to dentin, and it utilizes a self-etching primer with a pH of 2.7, which can be classified as “ultra-mild” [35]. There is currently no consensus about the bonding performance of the silorane adhesive system. Some studies found that silorane adhesive system showed equal performance with conventional methacrylate-based adhesives [36–38]. However our study is in line with the others that found lower bond efficiency [18, 39].

This study also found that, with the exception of FZ250-BFC, SBS values of laminated composites were not affected by the type of underlying material. Thus, the second null hypothesis tested was partially rejected. This is supported by the fact that adhesive failure was the most common failure type among FS specimens. Successful adhesion between dental materials is related to the chemical composition of their surfaces [35, 40]. The use of bulk-fill flowable composites is highly desired in routine restorative practice, as they can overcome the negative effects of polymerization shrinkage and can be placed and cured in a single layer of up to 4 mm in thickness [15]. While covering bulk-fill flowable composite material with a 2 mm layer of conventional composite resin has been recommended [23], there is limited knowledge regarding laminated usage of bulk-fill flowable composites. To our knowledge, this is the first study to compare the bond strengths of two different resin composites to a bulk-fill flowable composite and conventional underlying materials.

In line with previous studies [41–43], this study found the mean SBS value of FZ250 to RMGIC to be higher than that of FZ250 to CGIC, although the difference was not significant (*P* > 0.05). Some studies have suggested that the presence of HEMA and the formation of resin tags in RMGIC may be responsible for stronger bonding when compared to CGIC, regardless of the adhesive system used [44].

Failure analysis is a recommended method for assessing bonding of dental materials [45]. Cohesive failure is an indication that a strong bond has been formed at the interface of materials. In this study, not only was the bonding performance of FZ250 to BFC found to be better than to other underlying materials, cohesive failure was the main failure mode among FZ250-BFC specimens.

## 5. Conclusions 

Within the limitations of this study, the following can be concluded.

1. SBS values of FS are lower than SBS values of FZ250. For this reason, in cases with deep cavities or where there is questionable adaptation of resin composite to the cavity wall, incremental usage of FZ250 is preferable to FS.
2. Bulk-fill composite resin appears to be a suitable alternative for use as a base under methacrylate-based composite resins.

## Figures and Tables

**Table 1:** Study materials and application modes.

Materials	Contents	Application procedures	Lot number	Manufacturer
Filtek Z250	Bis-EMA (5%–10% by wt), silane-treated ceramic (75%–85% by wt), UDMA (5%–10% by wt), Bis-GMA (<5% by wt), TEGDMA (<5% by wt), water, zircon silica filler (60% by volume), and average particle size = 0.6 *μ*m	(1) Place in molds (2) Light-cure* for 20 s for a 2-mm-thick layer on one side	N452327	3M/ESPE, St. Paul, MN, USA

Filtek Silorane	3 Silorane (3,4-epoxycyclohexylethylcyclo polymethylsiloxane, bis-3,4-epoxycyclohexylethyl-phenylmethylsilane) Fillers: Quartz (silane layer), radiopaque yttrium fluoride filler (loading: 76% wt, 55% vol)	(1) Place in molds (2) Light-cure* for 20 s for a 2-mm-thick layer on one side	N426252	3M/ESPE, St. Paul, MN, USA

Clearfil SE bond primer	MDP, HEMA, and water	Dry gently with air for 5 s	041819	KURARAY, Tokyo, Japan

Clearfil SE bond adhesive	MDP, Bis-GMA, HEMA, hydrophobic dimethacrylate, and silanated colloidal silica	(1) Apply a thin layer for bonding (2) Gentle air blow (3) Light-cure* for 10 s	041819	KURARAY, Tokyo, Japan

Silorane self-etch primer	Phosphorylated methacrylates, Vitrebond copolymer, BisGMA, HEMA water, ethanol, silane-treated silica filler, initiators, and stabilizers	Dry gently with air for 5 s	N326885	3M ESPE Dental Products St. Paul, MN, USA

Silorane adhesive bond	Hydrophobie dimethacrylate, phosphorylated methacrylates, TEGDMA silane-treated silica filler, initiators, and stabilizer	(1) Apply a thin layer for bonding (2) Gentle air blow (3) Light-cure* for 10 s	N326930	3M ESPE Dental Products St. Paul, MN, USA

ÆliteFlo LV	Barium glass, colloidal silica monomers NA	(1) Place in molds (2) Light-cure* for 20 s on one side	1200007465	BISCO Inc.

Surefil SDR flow	SDR patented urethane dimethacrylate, dimethacrylate, ethoxylated bisphenol A dimethacrylate, pigment, photoinitiator, and barium and strontium alumino-fluoro-silicate glasses (68% wt, 45% vol)	(1) Place in molds (2) Light-cure* for 20 s on one side	081263	DENTSPLY

RİVA self-cure GIC	Fluoroaluminosilicate glass, polyacrylic acid, and tartaric acid	(1) Automatic mixing of capsules for 10 s (2) Hold in molds for 10 min	B1112091E6	SDI (Bayswater, VIC, AU)

GC Fuji II LC	Distilled water, polyacrylic acid, HEMA, urethane dimethacrylate, silicone dioxide, aluminosilicate glass, and urethane dimethacrylate	(1) Automatic mixing of capsules for 10 s (2) Light-cure* 20 s for 2 mm thick layer on one side	1204167	GC Corporation, Tokyo, Japan

BisEMA: bisphenol A ethoxylate dimethacrylate; BisGMA: bisphenol A glycidyl methacrylate; TEGDMA: triethylene dimethacrylate; UDMA: urethane dimethacrylate; MDP: methacryloyloxydecyl dihydrogen phosphate; HEMA: hydroxyethyl methacrylate; *Elipar free light II, 3M/ESPE, St. Paul, MN, USA; light intensity: 1000 mV/cm^2^.

**Table 2:** SBS values (MPa) and fracture surface analysis of FZ 250 and FS (*n* = 10).

Underlying material	FZ250 mean (SD)	Fracture analysis (A/M/C)	FS mean (SD)	Fracture analysis (A/M/C)
FLC	26.1 (2.6)^A.1^	2/3/5	14.0 (2.1)^A.2^	7/2/1
BFC	30.1 (2.5)^B.1^	1/3/6	13.9 (2.2)^A.2^	7/2/1
CGIC	22.3 (4.3)^A.1^	3/2/5	16.3 (4.3)^A.2^	6/1/3
RMGIC	24.8 (2.3)^A.1^	1/3/6	14.6 (4.4)^A.2^	8/1/1

Differences in superscript letters indicate statistically significant differences within columns, and differences in superscript numbers indicate statistically significant differences within rows (*P* ≤ 0.05).

For fracture surface analysis, A: adhesive failure, M: mixed failure, and C: cohesive failure.
